# The Impact of the First Wave of the COVID-19 Pandemic on Healthcare Workers: An Italian Retrospective Study

**DOI:** 10.3390/ijerph19095205

**Published:** 2022-04-25

**Authors:** Giuliana Buresti, Bruna Maria Rondinone, Diana Gagliardi, Marta Petyx, Fortunato Paolo D’Ancona, Patrizio Pezzotti, Flavia Riccardo, Sergio Iavicoli

**Affiliations:** 1 Department of Occupational and Environmental Medicine Epidemiology and Hygiene, Italian Workers’ Compensation Authority (INAIL), Via Fontana Candida 1, Monteporzio Catone, 00078 Rome, Italy; g.buresti@inail.it (G.B.); d.gagliardi@inail.it (D.G.); m.petyx@inail.it (M.P.); 2 Department of Infectious Diseases, Italian Institute of Health (ISS), Viale Regina Elena 299, 00161 Rome, Italy; paolo.dancona@iss.it (F.P.D.); patrizio.pezzotti@iss.it (P.P.); flavia.riccardo@iss.it (F.R.); 3 Directorate-General for Communication and European and International Relations, Italian Ministry of Health, Viale Giorgio Ribotta 5, 00144 Rome, Italy; s.iavicoli@sanita.it

**Keywords:** COVID-19, healthcare workers (HCWs), retrospective study, incidence

## Abstract

Healthcare workers (HCWs) played an essential role in managing the COVID-19 pandemic. The Italian Workers’ Compensation Authority (INAIL) and the Italian National Institute of Health (ISS) developed a retrospective study to analyze and understand trends and characteristics of infections among HCWs during the first wave of the pandemic. Between May and September 2020, Italian Regions retrospectively collected anonymous data regarding HCWs infected from the beginning of the pandemic until 30 April 2020 from their administrative sources through a questionnaire asking for socio-demographic and occupational information about the characteristics of contagion and disease outcome. Almost 16,000 valid questionnaires were received. Logistic regression was performed to ascertain the effect of age, gender, geographical macro area, profession, and pre-existing health conditions on the likelihood of HCWs developing more severe forms of COVID-19 (at least hospitalization with mild symptoms). All predictor variables were statistically significant. HCWs at higher risk of developing a more severe disease were males (OR: 1.90; 95% CI: 1.44–2.51), older than 60 years of age (OR: 6.00; 95% CI: 3.30–10.91), doctors (OR: 4.22; 95% CI: 2.22–9.02), working in Lombardy (OR: 55.24; 95% CI: 34.96–87.29) and with pre-existing health conditions (OR: 1.90; 95% CI: 1.43–2.51). This study analyses the main reasons for the overload put on the National Health Service by the first wave of the pandemic and the risk of infection for HCWs by age, gender, occupational profile and pre-existing health conditions. Improved knowledge, availability of personal protective equipment (PPE) and a tight vaccination campaign for HCWs strongly changed the trend of infections among HCWs, with substantial elimination of serious and fatal cases.

## 1. Introduction

In December 2019, a new coronavirus spread in Wuhan, Hubei Province, China, one of China’s largest cities with 11 million inhabitants [1,2]. This new virus, initially referred to as 2019-nCoV and later re-named SARS-CoV-2, caused Coronavirus Disease 2019 (COVID-19). On 30 January 2020, the WHO declared this disease a Public Health Emergency of International Concern (PHEIC) [3], and by 11 March 2020 declared COVID-19 a pandemic [4].

According to the WHO estimates [5], as of 20 March 2022, there are more than 468 million confirmed cases of COVID-19 globally, of which 32% are in the Americas and 41% are in Europe. Total deaths were more than 6 million, with 44% in the Americas and 32% in Europe.

Italy was the first among Western countries to face the spread of the pandemic and one of the most severely hit at the global level. The first case of COVID-19 was declared on 6 February 2020, and by 28 March 2022, there had been more than 14 million confirmed cases, with more than 156 thousand deaths [6].

COVID-19 has been recognized as a new occupational disease in several economic sectors in this scenario. Outbreaks were recorded among workers in meat and poultry processing facilities in the USA [7] and other countries, correctional and detention facilities [8] and homeless shelters [9]. In Italy, the impact of COVID-19 on workers may be measured by more than 229,000 compensation claims and 835 deaths related to occupational exposure to COVID-19 registered as of 28 February 2022 [10]. Among these, healthcare workers (HCWs) faced increasing exposure to the causal agent of COVID-19 [11,12,13,14]. The rapid growth in cases among the general population and the increased demand for healthcare made HCWs one of the occupational groups at the highest risk.

HCWs play an essential role in the health system. They have been directly involved in managing the epidemic, working at the forefront of patient care and ensuring that prevention and control measures are implemented to contain infections. They may be exposed to occupational hazards that put them at risk of disease and death in the management of COVID-19. These new occupational risks related to COVID-19 include not only occupational infections with COVID-19 but also skin disorders and heat stress due to the prolonged use of personal protective equipment (PPE); higher exposure to toxins due to increased use of disinfectants; psychological distress; increased fatigue; and stigma, discrimination, physical and psychological violence and harassment [15]. The size of the phenomenon and its sudden growth, unprecedented compared to the working population involved, entails very high costs in managing the health system’s sustainability, which has faced a new risk, completely unknown until two years ago, with all the related consequences.

On 8 April 2020, the WHO recorded more than 22,000 cases of COVID-19 among HCWs in 52 countries. However, as the organization itself pointed out, this figure might underestimate the real number [16] due to the absence of the systematic collection of data on infections in the health sector. In the United States, as of 28 March 2022, there were 1,067,910 HCWs infected. Death status was available for 701,772 (65.71%) cases, of whom 4075 died [17].

The crucial role of HCWs was recognized by the 73rd World Health Assembly held in November 2020, during which, through the decision WHA73(30), the year 2021 was designated as the International Year of Health and Care Workers [18].

In Italy, according to the COVID-19 integrated epidemiological surveillance run by the Italian National Institute of Health (ISS), between the beginning of the epidemic and 28 March 2022, more than 262,000 COVID-19 cases were recorded among HCWs [6], representing about 2.6% of the total cases in the general population [19]. However, the proportion of HCWs infected reached 12% of the total number of cases in the early stages of the pandemic [20,21], with a downward trend of the curve starting only in July–August 2020.

In Italy, the National Health Service is characterized by significant regional differences in organization and management strategies, resulting in various performance levels across regional health systems. Different regional protocols for managing COVID-19 emergencies may have contributed to the heterogeneity of outcomes observed between regions [14].

Based on this scenario and in the context of a wide synergy of joint initiatives implemented during the SARS-CoV-2 emergency, the Italian Workers’ Compensation Authority (INAIL), in collaboration with the ISS, has developed a retrospective study related to the first pandemic wave to analyse the trend and characteristics of infections among HCWs. 

This study aims to understand the dynamics of infections among HCWs from new and aggressive biological agents that can spread rapidly, causing epidemics, and identify a set of appropriate prevention and protection measures to put in place in case of similar future events. 

This paper will present a detailed description of the relevant literature and background information and detail the research methods and the statistical analysis performed. Finally, we will discuss the results, the limitations and the practical implications of the study.

## 2. Literature Review

Workers employed in the health sector are among those most severely hit by the pandemic due to their crucial role at the frontline of the health emergency; in addition, the spread of the SARS-CoV-2 infection contributed to the physical, mental, social well-being and emotional exhaustion of HCWs, with consequences on patient care [22,23].

As the first Western country to be affected by the pandemic, Italy has paid a high toll in terms of infections and deaths of HCWs due to the lack of preparedness to face such an unprecedented event [24]. During the first pandemic wave, patients with COVID-19 were added to patients with emergencies not related to COVID-19 and patients with essential routine needs. In addition to the regular workload (including emergency room and elective healthcare), HCWs had to cope with the burden of COVID-19 patients, which rapidly glutted the response capacities of the National Health Service. While the high rate of infections among HCWs resulted in a consequent lack of workers to face the pandemic [25], the parallel need to increase the number of intensive care unit (ICU) beds to accommodate all COVID-19 patients forced the reassignment of non-specialized HCWs (e.g., newly qualified students) to frontline positions to manage the pandemic [26,27]. Huy N.T. et al. [28] declared that COVID-19 awareness and preparedness among HCWs are vital to preventing transmission in healthcare settings. 

In addition, with sustained community transmission, HCWs could have close contact with patients presenting atypical, scarce or no disease symptoms [29]. It is well known that asymptomatic subjects contribute substantially to the spread of the virus [30,31,32]. However, in the first phases of the pandemic, due to the severe lack of swabs, only those patients with clear symptoms were tested, and this may have increased the number of unrecognized contacts in hospital wards or other healthcare settings with poor infection prevention and control (IPC) measures.

A typical example is nursing homes, where prevention measures are more difficult to apply due to the poor collaboration of patients (generally elderly, often disabled or with cognitive difficulties). PPE could be a barrier to their relationship with patients. To prevent infections among HCWs and reduce the spread of the virus, healthcare facilities designed periodical testing strategies for the early detection of new cases among HCWs [33]. 

In many countries, various studies have been developed to describe the characteristics of this phenomenon to put in place some measures to limit the spread of the virus. Most of these studies are described and synthesized in a living systematic review and meta-analysis [34], showing the prevalence, risk factors, clinical characteristics, and prognosis of SARS-CoV-2 infection among HCWs during the first wave of the pandemic. This study concluded that HCWs suffer a significant burden from COVID-19: it shows an 11% estimated prevalence of SARS-CoV-2 infection among HCWs, with those working in hospital non-emergency wards and nurses being the most infected personnel [34].

## 3. Research Methods

### 3.1. Research Approach

This is a retrospective cohort study. A retrospective study is an observational study that enrolls participants with a particular condition. It is a type of observational research in which the researcher looks back in time at archived or self-reported data using questionnaires, medical records, and other methods. This type of study aims to analyze data in detail to determine what potential risk factors or other associations and relationships exist within the participants. The ready availability of data (they have to be collected and analyzed) allows for lower costs, smaller sample sizes, and faster study completion. On the other hand, this method has some disadvantages, such as recall bias (a systematic error that occurs when participants do not remember previous events or experiences accurately or omit details) and missing data (important data may not have been collected) [35]. This type of research approach was selected for the present study to collect data regarding the contagion among the HCWs, highlighting the main critical issues in a brief period and reducing emergency costs due to COVID-19.

### 3.2. Development of the Survey Tool

In the present study, data were gathered retrospectively with the contribution of Regional Health Institutions through the involvement of key people identified in collaboration with the ISS in the context of the COVID-19 integrated epidemiological surveillance. Regions previously informed about the study’s aims were requested to collect data anonymously from their administrative sources regarding SARS-CoV-2 infections among HCWs from the beginning of the pandemic until 30 April 2020 through a questionnaire. A preliminary pilot test was conducted within a small group of key people to collect feedback on the clarity of questions and response options, sequence, flow, and data availability. Suggestions and observations provided by the pilot testers were included in developing the final version of the questionnaire.

The questionnaire consisted of three sections: socio-demographics, in which information regarding gender, age, and the province of residence were requested; occupational information, such as profession, type of work setting and type of employment; and infection details, including information on the date of diagnosis of COVID-19 and the course of the disease (hospitalization with mild symptoms or in ICU; discharge or death), being identified as “close contact” and in which context (occupational or not), and pre-existing health conditions. The questionnaire was paired with a brief description of the study and its aims. Data collection began on 6 May 2020, when the form was sent to regional institutions, and ended in September 2020. During this period, some reminders were sent to increase the response rate.

### 3.3. Study Subjects

An HCW represents the survey unit. For various reasons (including voluntary service), it comprises all individuals who carry out patient care activities, including doctors, nurses, socio-health workers, laboratory and radiology technicians. Professionals who are not directly involved in care activities, such as inpatient or outpatient admissions clerks, cashiers and service reservation clerks, have also been included.

### 3.4. Statistical Analysis

Statistical analysis was performed with IBM SPSS version 25.0 (IBM Corp. Released 2017. IBM SPSS Statistics for Windows, Version 25.0, IBM Corp., Armonk, NY, USA).

A descriptive analysis was carried out for some socio-demographic variables. Missing data were excluded if they were less than 5% of the total sample. 

Logistic regression was performed to ascertain the effect of age, gender, geographical macro area, profession, and pre-existing health conditions on the likelihood that HCWs would develop more severe forms of COVID-19 (at least hospitalization with mild symptoms). In the sample, information on the course of the disease (symptoms, hospitalization) was available for 5483 subjects. This analysis was performed in this latter subgroup, comparing asymptomatic HCWs versus HCWs with symptoms (at least hospitalization with mild symptoms). *p*-values < 0.05 were considered significant.

Information about the denominator was extracted from the Italian Institute of Statistics (ISTAT) tables of resident population and registry movement by region for 2019 [36] to calculate the incidence of COVID-19 for the general population. To calculate the incidence of COVID-19 for HCWs, data for the denominator were taken from the website of the Ministry of Health for the year 2019, the latest available at the time of the analysis [37]. Data for denominators included healthcare, administrative and technical workers.

We also tried to assess the impact of the healthcare system’s burden on HCW infections by comparing the number of total hospitalizations and admissions in intensive care in the general population (based on data collected through the COVID-19 integrated health surveillance conducted by the ISS) with data on HWC cases testing positive for COVID-19 and operating in the inpatient care hospitals collected for this study.

## 4. Results

### 4.1. Sample Description: Socio-Demographic and Professional Variables

We received 17,013 forms relating to cases registered between the beginning of the pandemic and 30 April 2020: 15,926 met all inclusion criteria; 1087 were diagnosed after the study cut-off date and therefore were excluded. Forms were received from seven Italian regions, with the highest percentages coming from Lombardy (63.7%) and Veneto (19.6%). The median age in the sample was 49 years (IQR = 33–65 years), and 10,691 cases (67.4%) involved females. The majority of HCWs were nurses (47.9%), followed by doctors (20.5%) and socio-health workers (19.7%). Females were more represented among socio-health workers (78.0%), nurses (76.5%) and administrative staff (70.9%), whereas males were most frequently doctors (58.1%) and technicians (51.7%). Most HCWs (88.7%) had a permanent contract, 3.4% had a fixed-term contract, and 7.9% had other types of contract (freelance, etc.). Table 1 describes the demographic and professional variables among HCWs with COVID-19 (*n* = 15,926) in Italy from 21 February to 30 April 2020.

### 4.2. Sample Description: Type of Facilities in Which HCWs Operate

A total of 76.5% of cases worked mainly in inpatient care facilities; almost all (94.2%) were hospitals, followed by social and health (4.2%) and “other” facilities (1.5%). Missing answers represented 11.4% of the data. Among hospitals, 72.4% were public health facilities, including local health authorities (ASL) and university hospitals or polyclinics, 14.0% were private care facilities, and 13.6% were Scientific Institutes for Inpatient Treatment and Research (IRCCS).

Among the social and health facilities, 29.4% were nursing homes (RSA), 19.5% were retirement homes, and 2.6% were hospices. However, it should be noted that about half (48.5%) of the subjects who declared that they worked in a social and health facility did not report further detailed information.

During the study period, 95.2% of health facilities at the national level had one or more wards converted for COVID-19 patients’ treatment. In Lombardy, 100.0% of structures had one or more wards converted to treat COVID-19 patients.

Most HCWs work in hospitals (78.0%) or local settings (13.3%). Administrative staff and workers employed in “other” fields account for less than 10% of the sample, respectively, 5.5% and 3.2%. Regarding the kind of hospital setting, the highest rate of contagions was recorded among HCWs working in medical wards (medical area) (21.2%); 15.6% of cases were recorded in wards dedicated or converted to COVID-19 treatment, and 10.2% were in surgical wards (Table 2).

### 4.3. Information Relating to Infections According to Age and Gender

Table 3 shows the number of cases, the number of hospitalizations with mild symptoms, the number of hospitalizations in the ICU and the number of deaths out of the total number of HCWs infected broken down by age and gender.

As of 30 April 2020, 3649 HCWs were hospitalized, of which 3633 were with mild symptoms (22.9% of the total sample) and 197 were in the ICU (1.2%); 63 deaths were recorded (0.4%). The same subject may have been registered in two or three states due to the progressive worsening of the disease. For 1854 subjects, information about the asymptomatic state is available. Considering the breakdown of age by class, we observed that the proportions of hospitalization with mild symptoms, ICU and death increase with age, reaching, in people over 60 years, 34.8%, 4.3%, and 2.1%, respectively. The disaggregation by gender shows the same trend; additionally, percentages of hospitalization with mild symptoms, ICU hospitalization and deaths were consistently higher in males for each age range. 

Being identified as a “close contact” was a piece of information available only for 953 subjects. Among these, in 52.5% of cases (500 subjects), the contact was in the family environment, in other types of context or not specified; in 47.5% of cases (453 subjects), the contact happened at the workplace, mainly with a patient (20.0%) or with other HCWs (15.7%). In comparison, for the remaining 11.8%, the source of contagion was not specified.

An in-depth analysis specifically referring to work settings showed that 40.9% of those working in hospital wards had a “close contact” in the family environment, in other types of context or not specified; the remaining 59.1% had a “close contact” in the work environment, mainly with patients (23.9%) and other HCWs (20.1%), while 15.1% had unspecified contacts. 

Instead, a “close contact” was mainly identified in the family environment (86.2%). The remaining 13.8% had a “close contact” in the work environment, mainly with patients (8.8%) and other HCWs (1.1%); 3.9% did not specify.

### 4.4. Information Relating to Pre-Existing Health Conditions

Table 4 shows the prevalence of pre-existing health conditions among HCWs. Cardiovascular diseases are the most represented (54.9% of infected HCWs). The breakdown of pre-existing health conditions by SARS-CoV-2 disease outcome (particularly for those who were asymptomatic or unspecified, hospitalized with mild symptoms, and hospitalized in intensive care and deaths) is also reported.

The logistic regression model was applied to the sample’s subgroup for which information on the level of severity of the disease was available (*n* = 5483), comparing asymptomatic HCWs (*n* = 1854) and HCWs that were at least hospitalized (*n* = 3649).

The logistic regression model was statistically significant, X^2^(13) = 4920.964, *p* < 0.001. The model explained 83.8% (Nagelkerke R^2^) of the variance in developing more severe outcomes of COVID-19 and correctly classified 94.0% of cases. Sensitivity was 93.6%, and specificity was 94.8%. The positive predictive value was 97.1%, and the negative predictive value was 89.0%. The area under the ROC curve was 0.972 (95% CI, 0.967 to 0.977), which is an outstanding level of discrimination, according to Hosmer et al. [38]. All predictor variables, age, gender, occupation, geographical macro area, and pre-existing health conditions were statistically significant (Table 5). Males had 1.90 times (95% CI: 1.44–2.51) higher odds of developing more severe outcomes than females. Older HCWs had an increased likelihood of developing more severe outcomes than younger HCWs, specifically for HCWs older than 60 (OR: 6.00; 95% CI: 3.30–10.91). HCWs with pre-existing health conditions had 1.90 times (95% CI: 1.43–2.51) higher odds of developing more severe outcomes than otherwise healthy HCWs. Regarding the occupation, doctors (OR: 4.22; 95% CI: 2.22–9.02), nurses (OR: 2.61; 95% CI: 1.43–4.80), socio-health workers (OR: 2.80; 95% CI: 1.48–5.31) and administrative staff (OR: 2.63; 95% CI: 1.26–5.49) had higher odds of developing more severe outcomes compared to the reference category. In the Northwest, HCWs had 55.24 times higher odds of developing more severe outcomes than HCWs in the South and the Islands (95% CI: 34.96–87.290.)

### 4.5. Incidence in the General Population and among HCWs

**Figure 1:**
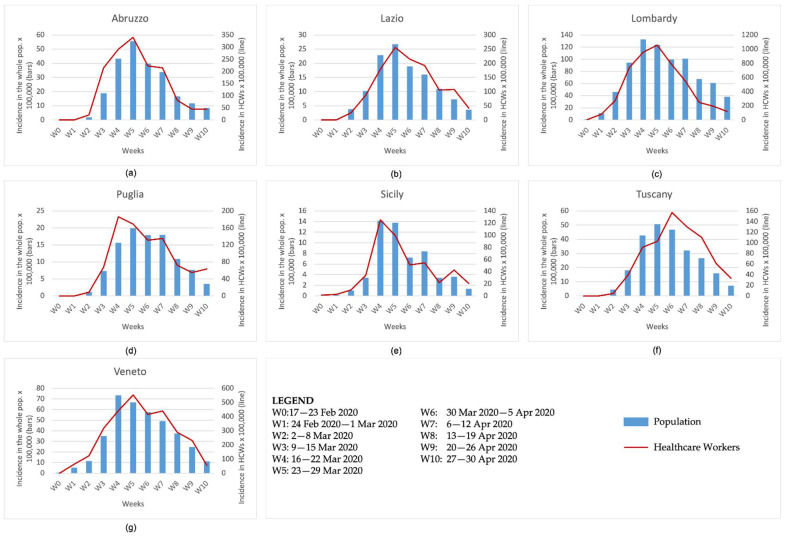
Incidence in the whole population and HCWs by week from the beginning of the pandemic until 30 April 2020 in (**a**) Abruzzo, (**b**) Lazio, (**c**) Lombardy, (**d**) Puglia, (**e**) Sicily, (**f**) Tuscany and (**g**) Veneto.

In Figure 1, the incidence in the whole population and HCWs for each region included in the study and for each week from the beginning of the pandemic until 30 April 2020 is shown. The incidence in the whole population is higher in Lombardy, where 132 cases per 100,000 inhabitants are observed in the fourth week (16–22 March 2020) since the pandemic, followed by Veneto, which counts in the same week about 70 cases per 100,000 inhabitants. These two regions also have the highest incidence among HCWs, with about 1000 and 550 cases per 100,000 HCWs. In addition, it is worth noting that the peaks of infections among HCWs have a one-week delay compared to the general population, being observed during the fifth week (23–29 March 2020) of the pandemic.

### 4.6. Number of Total Hospitalizations, Total Admissions in Intensive Care, and HCWs Tested Positive

**Figure 2:**
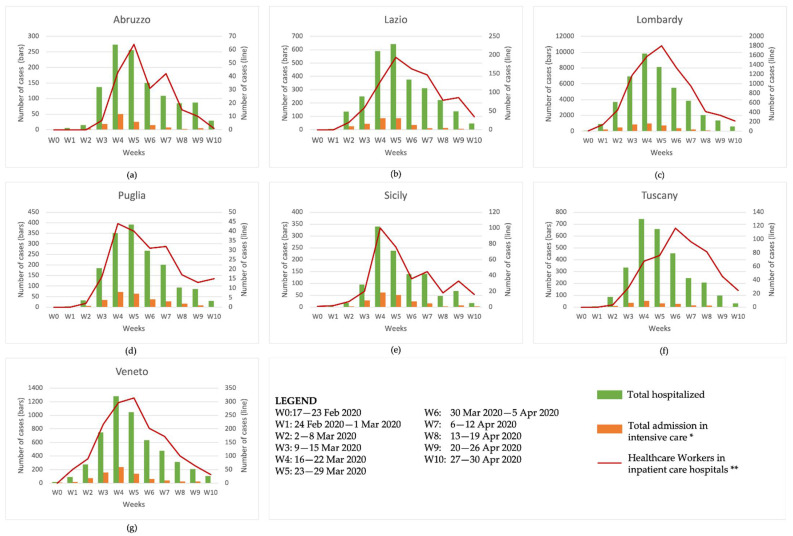
The number of total hospitalizations, total admissions in intensive care and HCWs in inpatient care hospitals by week from the beginning of the pandemic until 30 April 2020 in (**a**) Abruzzo, (**b**) Lazio, (**c**) Lombardy, (**d**) Puglia, (**e**) Sicily, (**f**) Tuscany and (**g**) Veneto. (*) ISS integrated epidemiological surveillance data; (**) retrospective study INAIL-ISS data.

Figure 2 shows the number of total hospitalizations and total admissions in intensive care as observed by the ISS COVID-19 integrated epidemiological surveillance data. We included HWC cases that tested positive for COVID-19 and operated in the inpatient care hospitals obtained in the same graphs. These data, presented for each region included in the study and for each week from the beginning of the pandemic until 30 April 2020, show that the regions with the highest number of hospitalizations, total admissions in intensive care, and HCWs who tested positive are Lombardy and Veneto. However, the peaks of hospitalizations and total admissions in intensive care were observed during the pandemic’s fourth week (16–22 March 2020), whereas the peak of HCWs who tested positive again had a one-week delay (23–29 March 2020).

## 5. Discussion

This study has analyzed the impact on Italian HCWs of the first wave of SARS-CoV-2 infection, which followed the appearance of a new and unknown viral agent and disease that led to an initial overburden of the healthcare system in terms of diagnosis, case tracking and management. Seven Italian regions (including Lombardy and Veneto, the most severely hit by the first pandemic wave) provided information regarding 15,926 HCWs who tested positive between the onset of the health emergency and 30 April 2020.

The study results show that most HCWs infected were females, but males had a higher risk of developing more severe disease. This is consistent with the socio-demographic and occupational profile within the health sector, where higher percentages of women are recorded among socio-health workers, nurses and administrative staff. In contrast, the proportion of men is higher among doctors. Most HCWs in our study were nurses, followed by doctors and socio-health workers with permanent contracts regarding the occupation. Concerning the outcome of the disease, our study showed that 22.9% of infected HCWs were hospitalized with mild symptoms, 1.2% were hospitalized in the ICU, and 0.4% died, with higher percentages for males in all three categories.

These figures are consistent with the result of our logistic regression model, which shows that males have an overall 1.90-fold risk of developing severe disease compared to females; among doctors, the risk is 4.22-fold compared to the reference category.

Our findings are consistent with the existing literature on COVID-19 infection and mortality among HCWs worldwide [39] as it demonstrates that infections are more frequent among females and nurses. At the same time, males and doctors have a higher burden of deaths. A deeper analysis of pre-existing health conditions showed that cardiovascular diseases are the most represented among infected HCWs; subjects with pre-existing health conditions had 1.90 higher odds of developing the more severe disease than otherwise healthy HCWs.

Even if the highest number of COVID-19 cases were reported in the 50–59 age range, the group aged over 60 years had the highest percentages of hospitalizations with mild symptoms, hospitalizations in ICU and deaths. According to the logistic regression, HCWs older than 60 years had 6.00 fold higher odds of developing the more severe disease than younger individuals. Additionally, these results align with that described in the literature [39]. 

The combined effect of age, gender and geographical distribution may play an important role in the pandemic diffusion. In the general population under 60 years of age, the number of COVID-19 cases is higher among women. This may be due to the higher percentage of women employed in jobs requiring closer contact with third parties (e.g., the healthcare sector and healthcare facilities) or delivering front-office services (e.g., cashiers, etc.). Moreover, different levels of urbanization may influence the spread of the virus due to the combination of various dimensions, such as population density, age, education, wealth, etc. These factors can jointly influence the vulnerability to the virus [40].

Our study shows that HCWs employed in inpatient care facilities were the most affected by the pandemic. Though widespread at a national level, this finding was particularly manifest in Lombardy and Veneto regions, where the infection and hospitalization curves among HCWs were very similar to that of the general population. 

Almost all inpatient health facilities had one or more wards dedicated to treating COVID-19 patients. The first phase of the pandemic overburdened the healthcare system, with impacts on local health settings at the community level, on care facilities and on hospital settings requiring a radical reorganization of wards and staff, including the conversion of many different wards into “COVID-19 departments” and the deployment of health workers not fully trained in infection prevention and control practices. In addition, the initial severe shortage of PPE may have contributed, at least partly, to the trend of the infection curve among HCWs, as they certainly had a higher risk of exposure to and contact with the viral agent.

Consistent with the literature [34], our study also revealed a higher incidence of cases in wards that traditionally do not manage infectious diseases or emergencies. In contrast, HCWs working in wards such as anesthesia, intensive care, emergency room or infectious diseases registered lower percentages of infections. Therefore, it can be assumed that a lack of training in IPC procedures and a possible lack of confidence in PPE donning and offing procedures may have played a role in the increased incidence of COVID-19 infections, at least in the first weeks of the epidemic.

Looking at the incidence and hospitalization curves, it must be noted that there was a one-week delay of the peak of contagion in both cases if compared with the trends in the general population. Most likely, this might be due to the impact of the burden of care on HCWs, especially at the beginning of the pandemic, when unacknowledged exposures to SARS-CoV-2 may have occurred.

### Limitations and Strengths

The study was meant to collect data and information from the entire national health system by inviting all 21 Italian regions to participate, but only 7 provided data. However, these included the most hit among these regions during the first wave of the pandemic—Lombardy and Veneto. The second limitation of our study is due to the nature of the data collected. Data provided by the different regions participating in the study were heterogeneous due to the different characteristics of the health services at the regional level and the adoption of different regional protocols for the management of COVID-19 emergencies [14]. To overcome this limitation, we made an initial effort to align data, especially those related to occupational information (e.g., wards, types of healthcare facility, etc.).

Despite these limitations, our study is the first in Italy to involve this number of subjects. Its results clearly show the characteristics of HCWs infected during the first wave of the pandemic and the difficulties the national health system encountered in managing the emergency. According to the data on characteristics of health staff units reported by ISTAT as of 31 December 2018 [41], our sample can represent the Italian health sector workforce. 

## 6. Conclusions

The results of our study can be considered a picture of the risk of COVID-19 among HCWs during the first wave of the pandemic. However, the comparison with data referring to the subsequent waves of the pandemic allows us to understand how the improvement of knowledge, increased testing and tracing capacities, widespread availability of PPE and a massive vaccination campaign, which was started by the end of December 2020 and is still ongoing, has strongly reduced the risk of exposure, thus changing the pattern of infection among HCWs. In particular, compulsory vaccination for HCWs facilitated a steep decrease in the contagion curve among HCWs, with a relevant drop in the proportion of cases among health professionals compared to the total number of cases [19]. Moreover, future studies will confirm nurses and HCWs working in non-emergency wards as the most frequently affected subjects. In that case, policymakers will be able to take advantage of some critical information to manage and contain viral transmission in healthcare environments. 

Finally, age, gender, type of work, and pre-existing health conditions influence the spread of the pandemic. For this reason, data collection should always include detailed socio-demographic, geographical and professional information to address the preparedness and response actions in healthcare facilities and to define targeted policy intervention. Further comparative analyses on the trends of infections among HCWs in subsequent waves of the pandemic may enable us to also quantitatively assess the impact of the prevention and protection measures that have been progressively adopted. Indeed, the experience achieved during these two years of the pandemic allowed the understanding of major gaps and needs and preparedness and response interventions to be put in place if an outbreak of new and unknown biological agents should happen again. The readiness and intensive use of appropriate prevention measures, including IPC training, availability of PPE and timely vaccination, are the most important recommendations for protecting HCWs. 

## Figures and Tables

**Table 1:** Demographic and occupational variables among HCWs with COVID-19 (*n* = 15,926) in Italy, 21 February–30 April 2020.

Variables (No. with Available Information)	*n*	%
Sex (15,863)		
Female	10,691	67.4
Male	5172	32.6
Age group (years) (15,924)		
18–29	1395	8.8
30–39	2569	16.1
40–49	4376	27.5
50–59	5531	34.7
≥60	2053	12.9
Geographical macro area (15,926)		
Northwest (Lombardy)	10,140	63.7
Northeast (Veneto)	3118	19.6
Center (Lazio, Tuscany)	1713	10.8
South and Islands (Abruzzo, Puglia, Sicily)	955	6.0
Type of contract (10,891)		
Permanent contract	9663	88.7
Fixed-term contract	365	3.4
Other type	863	7.9
Profession (15,413)		
Nurses	7373	47.9
Doctors	3150	20.5
Socio-health workers	3046	19.7
Administrative personnel	936	6.1
Other	908	5.9

**Table 2:** Distribution by area of activity with details of hospital context.

Department/Area	Area of Activity	% of HCWs Infected
Medical Area	Hospital setting	21.2
COVID-19 Units	Hospital setting	15.6
Territorial Assistance	Local setting	13.3
Surgical Area	Hospital setting	10.2
Rehabilitation and Prostheses	Hospital setting	8.5
Administration Area	Administrative Area	5.5
Anesthesia, Intensive Care and Emergency	Hospital setting	4.9
Infectious Disease	Hospital setting	4.6
Emergency and Urgent Care	Hospital setting	4.3
Diagnostic and Laboratory	Hospital setting	4.3
Pediatric Area (Medicine and Surgery)	Hospital setting	2.7
Outpatient/Hospital Outpatient Clinic	Hospital setting	1.6
Other	Other	3.2

**Table 3:** The number of total cases and percentages of HCWs hospitalized with mild symptoms, hospitalized in intensive care and deaths in total cases, by gender and class of age.

Class of Age	Number of Cases	Hospitalized with Mild Symptoms *n* (% on Cases)	Hospitalized in Intensive Care *n* (% on Cases)	Deaths *n* (% on Cases)
Males				
18–29	424	55 (13.0%)	0 (0.0%)	0 (0.0%)
30–39	868	155 (17.9%)	3 (0.3%)	0 (0.0%)
40–49	1161	279 (24.0%)	15 (1.3%)	2 (0.2%)
50–59	1518	495 (32.6%)	52 (3.4%)	11 (0.7%)
≥60	1200	492 (41.0%)	77 (6.4%)	36 (3.0%)
Total	5171	1476 (28.5%)	147 (2.8%)	49 (0.9%)
Females				
18–29	968	108 (11.2%)	1 (0.1%)	0 (0.0%)
30–39	1691	292 (17.3%)	3 (0.2%)	1 (0.0%)
40–49	3191	634 (19.9%)	13 (0.4%)	1 (0.0%)
50–59	3995	904 (22.6%)	23 (0.6%)	6 (0.2%)
≥60	846	219 (25.9%)	10 (1.2%)	6 (0.8%)
Total	10,691	2157 (20.2%)	50 (0.5%)	14 (0.1%)
Total				
18–29	1392	163 (11.7%)	1 (0.0%)	0 (0.0%)
30–39	2559	447 (17.5%)	6 (0.2%)	1 (0.0%)
40–49	4352	913 (21.0%)	28 (0.6%)	3 (0.1%)
50–59	5513	1399 (25.4%)	75 (1.4%)	17 (0.3%)
≥60	2046	711 (34.8%)	87 (4.3%)	42 (2.1%)
Total	15,862	3633 (22.9%)	197 (1.2%)	63 (0.4%)

**Table 4:** Pre-existing health conditions in the total sample and according to the disease outcome. Multiple-choice question ^1^.

Pre-Existing Health Conditions	Total	Asymptomatic/ Unspecified	Hospitalized with Mild Symptoms	Hospitalized in Intensive Care	Deaths
Cardiovascular diseases	54.9%	51.3%	64.7%	70.0%	80.6%
Oncological pathology	17.4%	17.2%	18.2%	15.0%	12.9%
Respiratory diseases	14.7%	13.5%	17.9%	10.0%	3.2%
Dysmetabolic disorders	14.1%	12.5%	18.4%	22.5%	38.7%
Other diseases	15.8%	18.4%	8.9%	10.0%	9.7%
None of the previous mentioned	6.7%	7.2%	5.4%	3.8%	3.2%

^1^ Percentage of cases are reported. Number of cases = 4026; number of responses = 4980.

**Table 5:** Logistic regression predicting the likelihood of a severe disease outcome based on age, gender, geographical macro area and occupation.

	B	SE	Wald	df	*p*	OR	95% CI	
							Lower	Upper
Age								
18–29						Ref		
30–39	0.63	0.30	4.38	1	0.036	1.87	1.04	3.37
40–49	1.02	0.28	13.72	1	<0.001	2.78	1.62	4.78
50–59	1.31	0.27	22.84	1	<0.001	3.69	2.16	6.31
≥60	1.79	0.31	34.42	1	<0.001	6.00	3.30	10.91
Gender								
Female						Ref		
Male	0.64	0.14	20.25	1	<0.001	1.90	1.44	2.51
Geographical macro area								
South and Islands						Ref		
Center	3.38	0.32	113.34	1	<0.001	29.33	15.75	54.63
Northwest	4.01	0.23	295.37	1	<0.001	55.24	34.96	87.29
Northeast	−2.74	0.19	218.89	1	<0.001	0.065	0.045	0.093
Profession								
Other						Ref		
Doctors	1.44	0.33	19.23	1	<0.001	4.22	2.22	9.02
Nurses	0.96	0.31	9.62	1	0.002	2.61	1.43	4.80
Socio-health workers	1.03	0.33	10.03	1	0.002	2.80	1.48	5.31
Administrative	0.97	0.38	6.67	1	0.010	2.63	1.26	5.49
Pre-existing health conditions								
No						Ref		
Yes	0.64	0.14	20.26	1	<0.001	1.90	1.43	2.51

## Data Availability

Not applicable.
